# O049. Psychodynamic functioning in chronic headache patients: a short term psychodynamic psychotherapy (STPP) study

**DOI:** 10.1186/1129-2377-16-S1-A105

**Published:** 2015-09-28

**Authors:** Barbara Petolicchio, Martina Squitieri, Alessandro Viganò, Massimiliano Toscano, Arianna Sirolli, Sara Aielli, Romina Di Giambattista, Edoardo Vicenzini, Edmond Gilliéron, Vittorio Di Piero

**Affiliations:** Department of Neurology and Psychiatry, Sapienza University of Rome, Rome, Italy; Istituto Europeo di Psicoterapia Psicoanalitica (IREP) of Rome, Rome, Italy

## Background

Chronic headache (CM) occurs in 2-5% of the general population, often associated with medication-overuse headache (MOH), and comorbid psychiatric disorders[1, 2]. Among therapeutic approaches, psychotherapeutic interventions may be effective, either alone or associated with pharmacological therapies. As we previously showed, the short-term psychodynamic psychotherapy (STPP), plus drug therapy, is more effective in patients with probable MOH to reduce headache symptoms and relapse rate than drug therapy alone[3]. Moreover, STPP alone is not inferior to valproate in CM, as preventive therapy[4]. According to psychodinamic diagnosis (BPI) some psychodynamic profiles with poor ability to process the emotional content or low mentalizing level (i.e., pre-psychosis, psychosis and borderline) could be at risk of developing chronic headaches. The aim of the present study was to identify the most frequent psychodynamic profiles in CM and test the effective of STPP in those patients with no record of psychiatric disorders.

## Methods

We consecutively recruited all CM patients, with or without MOH, attending our Headache Clinic over two years, according to the ICHD-II criteria. The protocol of psychotherapy reckoned on a first evaluation with 4 Brief Psychodynamic Investigation (BPI) and then psychotherapy treatment over the subsequent 2 months. At baseline, all patients with MOH were instructed to withdraw from the abused drugs. Follow-ups were planned at 15, 30 and 60 days when headache clinical features were recorded. HIT6, MIDAS and Depression and Anxiety Hamilton scales were also acquired.

## Results

We recruited 105 patients with chronic migraine (74% with MOH). Forty-eight patients (46%) did not complete the protocol. Fifty-seven patients (54%) actively participated in the study. According to BPI criteria, the patients were diagnosed as “psychotic” (44%), “pre-psychotic” (28%) and “borderline” type (28%). Clinically, 40% (n=23) of patients completed the full treatment period with a significant improvement of disease parameters (33% less attack duration, 17% less pain intensity, 41% lowering in MIDAS score, and 93% less medication overuse). However, we did not observe any correlation between headache characteristics and psychodymanic profiles.

## Conclusions

This study suggested that CM, with or without MOH, is associated with a low mentalizing level, condition characterized by a poor ability to process the emotional content. We confirm that the short term psychodynamic psychotherapy is effective in the treatment of CM.

Written informed consent to publication was obtained from the patient(s).
